# Cytokines in primary headache disorders: a systematic review and meta-analysis

**DOI:** 10.1186/s10194-023-01572-7

**Published:** 2023-04-04

**Authors:** Abdu Kisekka Musubire, Sanjay Cheema, Jason C. Ray, Elspeth J. Hutton, Manjit Matharu

**Affiliations:** 1 University College London (UCL) Queen Square Institute of Neurology, London, UK; 2 Infectious Diseases Institute, College of Health Sciences, Makerere University, Kampala, Uganda; 3 Kiruddu National Referral Hospital, Kampala, Uganda; 4 The National Hospital for Neurology and Neurosurgery, Queen Square, London, UK; 5 Department of Neurology, Alfred Health, Melbourne, Australia; 6 Department of Neuroscience, Monash University, Melbourne, Australia; 7 Department of Neurology, Austin Health, Melbourne, Australia

**Keywords:** Migraine disorders, Tension-type headache, Cytokine, Neurogenic inflammation, Immunology

## Abstract

**Background:**

The role of inflammation and cytokines in the pathophysiology of primary headache disorders is uncertain. We performed a systematic review and meta-analysis to synthesise the results of studies comparing peripheral blood cytokine levels between patients with migraine, tension-type headache, cluster headache, or new daily persistent headache (NDPH), and healthy controls; and in migraine between the ictal and interictal stages.

**Methods:**

We searched PubMed/Medline and Embase from inception until July 2022. We included original research studies which measured unstimulated levels of any cytokines in peripheral blood using enzyme-linked immunosorbent assay or similar assay. We assessed risk of bias using the Newcastle–Ottawa Quality Assessment Scale. We used random effects meta-analysis with inverse variance weighted average to calculate standardised mean difference (SMD), 95% confidence intervals, and heterogeneity for each comparison. This study is registered with PROSPERO (registration number CRD42023393363). No funding was received for this study.

**Results:**

Thirty-eight studies, including 1335 patients with migraine (32 studies), 302 with tension-type headache (nine studies), 42 with cluster headache (two studies), and 1225 healthy controls met inclusion criteria.

Meta-analysis showed significantly higher interleukin (IL)-6 (SMD 1.07, 95% CI 0.40–1.73, *p* = 0.002), tumour necrosis factor (TNF)-α (SMD 0.61, 95% CI 0.14–1.09, *p* = 0.01), and IL-8 (SMD 1.56, 95% CI 0.03–3.09, *p* = 0.04), in patients with migraine compared to healthy controls, and significantly higher interleukin-1β (IL-1β) (SMD 0.34, 95% CI 0.06–0.62, *p* = 0.02) during the ictal phase of migraine compared to the interictal phase. Transforming growth factor (TGF)-β (SMD 0.52, 95% CI 0.18–0.86, *p* = 0.003) and TNF-α (SMD 0.64, 95% CI 0.33–0.96, *p* = 0.0001) were both higher in patients with tension-type headache than controls.

**Conclusions:**

The higher levels of the proinflammatory cytokines IL-6, IL-8 and TNF-α in migraine compared to controls, and IL-1β during the ictal stage, suggest a role for inflammation in the pathophysiology of migraine, however prospective studies are required to confirm causality and investigate the mechanisms for the increase in cytokine levels identified. Cytokines may also have a role in tension-type headache. Due a lack of data, no conclusions can be made regarding cluster headache or NDPH.

## Background

The pathophysiology of the primary headache disorders tension-type headache, migraine, cluster headache, and new daily persistent headache (NDPH), and the degree of overlap in pathophysiology between the disorders, is incompletely understood [1–4].

Cytokines are small proteins important in cell signaling, particularly in regulation of the immune system. They have complex mechanisms of action but are broadly classified as pro-inflammatory, such as interleukin (IL)-6 and tumour necrosis factor alpha (TNF-α); or anti-inflammatory, such as IL-4 and IL-10 [5, 6].

Several observations suggest that the neuroinflammation and pro-inflammatory cytokines, may be involved in the pathophysiology of episodic headache attacks. These observations include the high frequency of acute headache as a symptom of systemic infection such as influenza and Covid-19 which may be mediated by cytokines [7–9], the high frequency of headache as an adverse effect of the therapeutic administration of the cytokines TNF-α or beta interferon for cancer or multiple sclerosis [10, 11], and the efficacy of non-steroidal anti-inflammatory drugs in acute treatment of headache attacks [12].

Cytokines and neurogenic inflammation are also hypothesised to be involved in the process of transformation from an episodic headache disorder to chronic daily headache, and/or in the development of de-novo chronic daily headache in post-infectious NDPH [9, 13, 14].

Several studies have measured serum levels of cytokines in patients with primary headache disorders, either making a comparison between patients with primary headache disorders and healthy controls, or between the ictal and interictal periods of migraine. We sought to synthesise the results of these studies to better determine the significance of cytokines in primary headache disorders.

### Objectives

To perform a systematic review and meta-analysis of studies measuring peripheral blood cytokine levels in primary headache disorders. Specifically, to determine whether there are differences in cytokine levels between the following groups:

- Patients with migraine compared to healthy controls
- During the ictal phase of migraine compared to the interictal phase
- Patients with tension-type headache compared to healthy controls
- Patients with cluster headache compared to healthy controls
- Patients with new daily persistent headache compared to healthy controls

## Methods

This systematic review and meta-analysis was conducted and reported according to Preferred Reporting Items for Systematic Reviews and Meta-Analyses (PRISMA) 2020 statement [15]. It is registered with PROSPERO (registration number CRD42023393363). The protocol was not pre-published. No funding was received for this review.

### Search strategy

Two reviewers (AKM and SC) independently searched the databases PubMed and Embase, with no start date and last search date on 7^th^ July 2022. Search terms were or “migraine” or “tension-type headache” or “cluster headache” or “trigeminal autonomic cephalalgia(s)” or “new daily persistent headache” or “primary headache” and “cytokine(s)”. The result was limited to humans as an automated filter. Duplicates and non-English language articles were removed before screening.

### Selection criteria

Titles and abstracts were screened for relevance by a single author (AKM), and full text was reviewed if the abstract indicated that cytokines were measured in any of the primary headaches under investigation. Full texts were then screened against the inclusion criteria. We included original research studies which included patients with migraine, tension-type headache, cluster headache, and/or new daily persistent headache according to International Headache Society (IHS) criteria; and which measured levels of any cytokines in peripheral blood using enzyme-linked immunosorbent assay (ELISA) or similar assay. We excluded any study where cytokine production was stimulated, any study where headache attacks were pharmacologically provoked, or interventional studies where baseline cytokine levels were not reported prior to intervention. In addition, we excluded any study where mean/standard deviation or median/IQR could not be extracted from the text, tables, or graphs.

### Data extraction

Data were extracted manually from included studies by a single author (AKM) into a Microsoft Excel spreadsheet. Data in this paper are presented as means and standard deviations. If only standard error of the mean (SEM) was reported, this was converted to standard deviation (SD). If only median and interquartile range (IQR) were reported, the median was assumed mean and the IQR converted to SD.

### Risk of bias assessment

Risk of bias was assessed by a single author (AKM) using the Newcastle–Ottawa Quality Assessment Scale [16].

### Statistical analysis

Data on demographics and study types were analysed using descriptive statistics in Microsoft Excel. Meta-analysis was performed where at least two studies had examined a particular cytokine for each of the group comparisons of interest. We used random effects meta-analysis with inverse variance weighted average, using the software RevMan 5 (Cochrane Collaboration). As all studies did not use the same ELISA-based methods for measurement of cytokines or report the same measurement units, standardised mean difference (SMD) was calculated for comparison of group differences and calculation of 95% confidence intervals. The I^2^ measure of heterogeneity was calculated for each meta-analysis. Funnel plots were used to assess for publication bias for those meta-analyses which included at least ten studies.

## Results

**Fig. 1:**
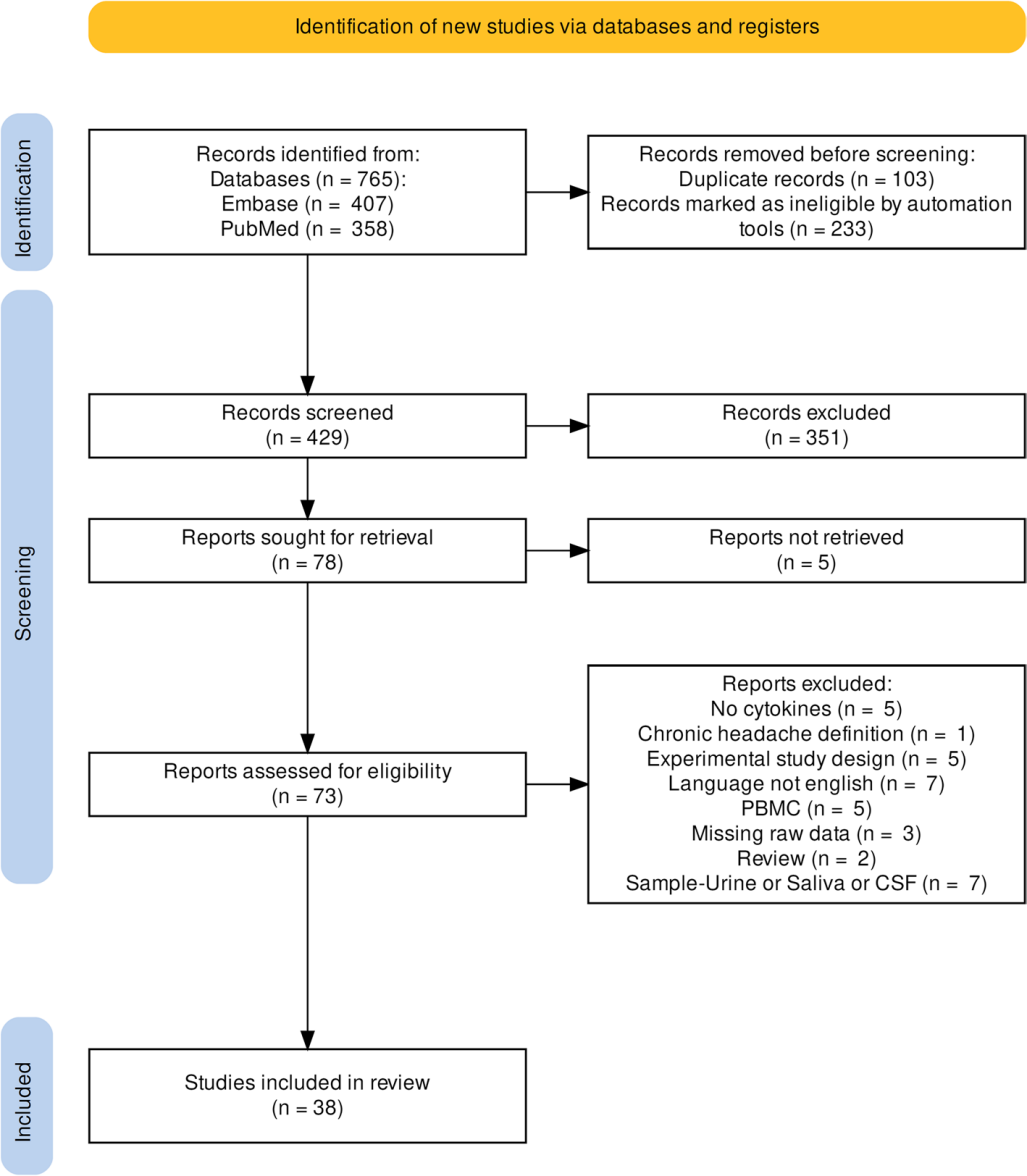
PRISMA flow diagram of study selection. Abbreviations: PMBC, studies measuring cytokine production by peripheral blood mononuclear cells in vitro, rather than circulating serum cytokines

The search resulted in 765 records, of which 38 met inclusion criteria (see Fig. 1). The studies included a total of 2904 participants, of whom 1335 had migraine, 302 had tension-type headache, 42 had cluster headache, and 1225 were healthy controls. No studies including patients with NDPH met inclusion criteria.

Of the 38 studies, 32 included patients with migraine, nine with tension-type headache, two with cluster headache, and none with new daily persistent headache. All studies used IHS criteria, most commonly ICHD-2 (17 studies) and ICHD-3 (11 studies) [17, 18]. Most (26/38) studies were conducted in outpatients. The most commonly measured cytokines were IL-6 (21 studies), TNF-α (20 studies), IL-10 (12 studies), IL-1β (11 studies), and IL-8 (10 studies). Study quality was rated as good (7 or 8 on Newcastle–Ottawa scale) in 31/38 studies. Details of individual studies are shown in Table 1.

**Table 1:** Summary of individual studies and quality assessment scores

Author	Year	Country	Condition(s) studied	ICHD criteria	Setting	Cytokines studied	NOS
Covelli et al. [19]	1991	Italy	M & T	1	NR	TNF-α	4
Shimomura et al. [20]	1991	Japan	M & T	I	OP	IL-2	7
Martelletti et al. [21]	1993a	Italy	C	I	OP	IL-1β	8
Martelletti et al. [22]	1993b	Italy	M	I	IP	IL-4, IL-6 IFN-γ	8
Munno et al. [23]	1998	Italy	M	I	OP	IL-4, IL-8, IFN-γ	8
Martelletti et al. [24]	2001	Italy	M	I	NR	IL-2, IL-4, IFN-γ	7
Empl et al. [25]	2003a	Germany	C	I	OP	IL-1β, IL-6	7
Empl et al. [26]	2003b	Germany	M	I	OP	IL-6, TNF-α	7
Sarchielli et al. [27]	2004	Italy	M	II	IP	IL-8	8
Perini et al. [28]	2005	Italy	M	I	OP	IL-1β, IL-2, IL-4, IL-6, IL-10, TNF-α	8
Ishizaki et al. [29]	2005	Japan	M & T	I	OP	TGF-β	8
Sarchielli et al. [30]	2006	Italy	M	II	IP	IL-1β, IL-4, IL-6, TNF-α	8
Fidan et al. [31]	2006	Turkey	M	II	IP	IL-6, IL-10	7
Koçer et al. [32]	2009	Turkey	M	II	OP	IL-6	6
Bockowski et al. [33]	2009	Poland	M & T	II	IP	IL-1α, TNF-α	8
Koçer et al. [34]	2010	Turkey	T	II	OP	IL-8	8
Bockowski et al. [35]	2010	Poland	M & T	II	IP	IL-4, IL-10, IL-13	5
Uzar et al. [36]	2011	Turkey	M	II	IP	IL-1β, IL-2, IL-6, IL-10, TNF-α	7
Güzel et al. [37]	2013	Turkey	M	II	OP	TGF-β	6
Della Vidova et al. [38]	2013	Australia	T	II	OP	IL-2, IL-5, IL-10, IL-13, I IFN-γ, TGF-β, TNF-α	8
Wang et al. [39]	2015	China	M	II	OP	IL-6	8
Lee et al. [40]	2015	Taiwan	M	III	OP	IL-1β, IL-2, IL-4, IL-6, IL-8, IL-10	8
Duarte et al. [41]	2015	Brazil	M	II	OP	IL-8	8
Domingues et al. [42]	2015	Brazil	T	II	OP	IL-8	6
Deitos et al. [43]	2015	Brazil	T	II	OP	IL-8, IL-12, TNF-α	5
Aydin et al. [44]	2015	Turkey	M	III	NR	IL-4, IL-5, IL-6, IL-10, IFN-γ, TNF-α	7
Yucel et al. [45]	2016	Turkey	M	II	IP	IL-1β, IL-6, TNF-α	7
Oliveira et al. [46]	2017	Brazil	M	II	OP	IL-6, IL-8, IL-10, TNF-α	8
Michalak et al. [47]	2017	Poland	M	II	OP	TNF-α	8
Martami et al. [48]	2018	Iran	M	III	OP	IL-6, TNF-α	7
Dominguez et al. [49]	2018	Spain	M	III	OP	IL-6, IL-10, TNF-α	8
Han et al. [50]	2019	China	M	III	OP	IL-1β, IL-2, IL-6, IL-10, TNF-α	7
Flook et al. [51]	2019	Spain	M	III	OP	IL-1α, IL-1β, IL-4, IL-6, IL-8, IFN-γ	3
Chaudhry et al. [52]	2019	Germany	M	III	NR	IL-1β, IL-6, IL-10, TNF-α	8
Togha et al. [53]	2020	Iran	M	III	OP	IL-6, TNF-α	8
Karaaslan et al. [54]	2020	Turkey	M	III	OP	IL-1β, IL-6, TNF-α	7
Dönder et al. [55]	2021	Turkey	M	III	OP	IL-18	8
Cowan et al. [56]	2021	USA	M	III	OP	IL-6, IL-8, IL-10, IFN-γ, TNF-α	6

IL-1α, IL-5, IL-12 and IL-13 were not used for meta-analysis as they were only reported in single studies for any of the comparisons of interest

*Abbreviations*: *C* Cluster headache, *ICHD* International Classification of Headache Disorders, *IP* Inpatient, *M* Migraine, *NOS* Newcastle–Ottawa score, *NR* Not recorded, *OP* Outpatient, *T* Tension-type headache

### Migraine versus healthy controls

**Fig. 2:**
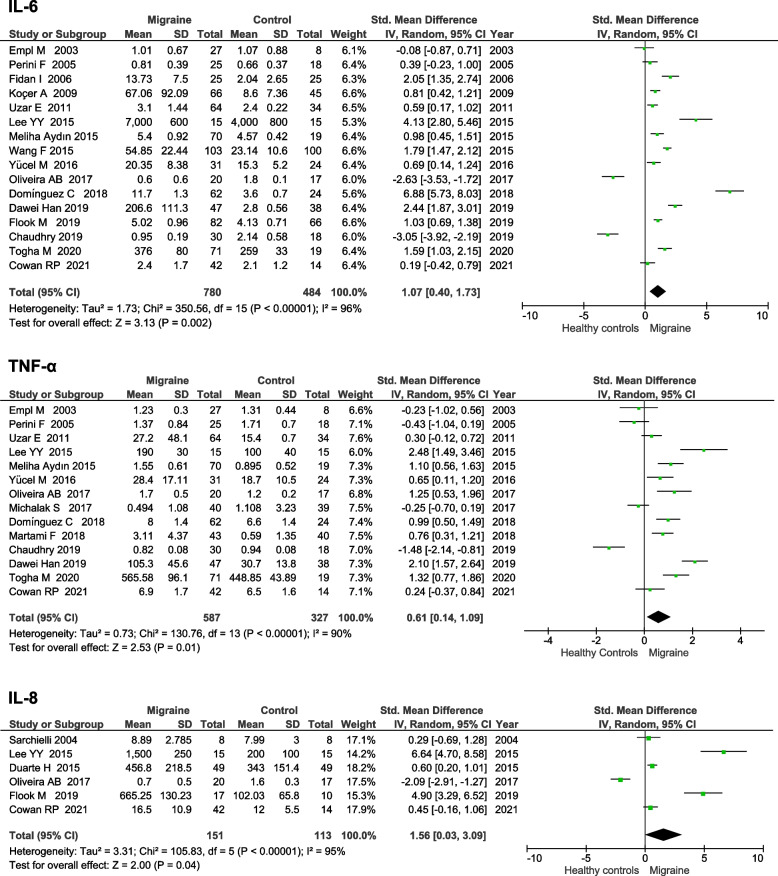
Forest plots of IL-6, TNF-α, and IL-8 levels in migraine compared to healthy controls. Abbreviations: IL, interleukin; IV, inverse variance; I^2^, measure of heterogeneity; SD, standard deviation; TNF-α, tumour necrosis factor alpha

**Fig. 3:**
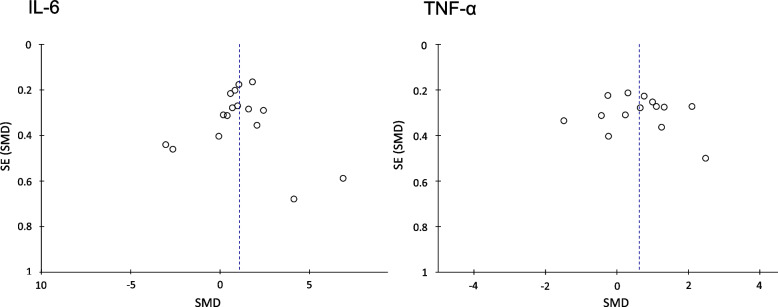
Funnel plots for studies of IL-6 and TNFα in migraine compared to healthy controls. Abbreviations: IL, interleukin; TNF-α, tumour necrosis factor alpha; SD, standard deviation; SEM, standard error of the mean

Nine cytokines were assessed in at least two studies comparing migraine to healthy controls (see Table 2). The two most commonly measured cytokines (IL-6 and TNF-α) were both higher in migraine than controls. IL-6 had a SMD of 1.07 (95% CI 0.40–1.73, *p* = 0.002) and TNF-α had a SMD of 0.61 (95% CI 0.14–1.09, *p* = 0.01). IL-8 was also higher in migraine than healthy controls (SMD 1.56 95% CI 0.03–3.09, *p* = 0.04). Forest plots for IL-6, TNF-α, and IL-8 are displayed in Fig. 2. Funnel plots for IL-6 and TNF-α did not show evidence of publication bias (See Fig. 3), funnel plot for IL-8 was not generated as there were fewer than ten studies included. There were no significant differences in the other cytokines measured (see Table 2). Heterogeneity levels were high for all cytokines in the comparison of migraine and healthy controls.

**Table 2:** Differences in circulating cytokines levels between patients with migraine and healthy controls

Cytokine	N of studies	Total participants	Std. Mean Difference (95% CI) Effect Estimate	*P* Value	I^2^
IL-1β	7	502	0.50 (-0.54, 1.54)	0.34	96%
IL-2	6	348	0.58 (-0.37, 1.54)	0.23	92%
IL-4	5	346	0.88 (-1.65, 3.41)	0.49	98%
IL-6	16	264	1.07 (0.40, 1.73)	0.002	96%
IL-8	6	163	1.56 (0.03, 3.09)	0.04	95%
IL-10	12	706	0.10 (-0.74, 0.94)	0.41	95%
TGF-β	2	219	2.05 (-0.29, 4.40)	0.09	98%
TNF-α	14	914	0.61 (0.14, 1.09)	0.01	90%
IFN-γ	6	301	1.23 (-0.03, 2.49)	0.06	93%

*Abbreviations*: *CI* Confidence interval, *I*^*2*^ Measure of heterogeneity, *IL-1β* Interleukin-1β, *IL-*, Interleukin-2, *IL-6* Interleukin-6, *IL-4* Interleukin-4, *IL-8* Interleukin-8, *IL-10* Interleukin-10, *TGF-β* Transforming growth factor β, *TNF-α* Tumor necrosis factor alpha, *IFN-γ* Interferon gamma

### Migraine ictal phase versus interictal phase

**Fig. 4:**
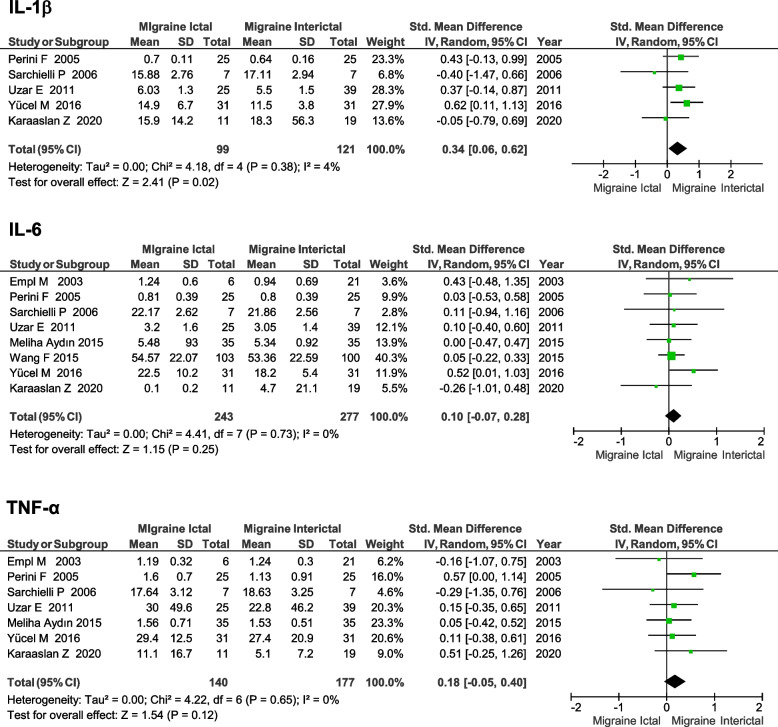
Forest plot of IL-1β, IL-6 and TNF-α levels in patients with migraine in the ictal stage compared to interictally. Abbreviations: IL, interleukin; IV, inverse variance; I^2^, measure of heterogeneity; SD, standard deviation

Six cytokines were assessed in at least two studies comparing the ictal phase of migraine to the interictal stage (see Table 3). IL-1β was higher in the ictal stage (SMD 0.34, 95% CI 0.06–0.62, *p* = 0.02), based on five studies with a low degree of heterogeneity (I^2^ = 4%) (see Fig. 4). There were no significant differences in the other cytokines measured (see Table 3).

**Table 3:** Differences in circulating cytokines levels in the ictal stage of migraine compared to the inter-ictal stage

Cytokine	N of studies	Total participants	Std. Mean Difference (95% CI) Effect Estimate	*P* Value	I^2^
IL-1β	5	220	0.34 (0.06, 0.62)	0.02	4%
IL-2	2	114	-0.09 (-0.89, 0.70)	0.82	77%
IL-4	4	174	-0.24 (-0.90, 0.42)	0.48	76%
IL-6	8	520	0.10 (-0.07, 0.28)	0.25	0%
IL-10	3	184	0.23 (-0.23, 0.68)	0.33	57%
TNF-α	7	317	0.18 (-0.05, 0.40)	0.12	0%

*Abbreviations*: *CI* Confidence interval, *I*^*2*^ Measure of heterogeneity, *IL-2* Interleukin-2, *IL-8* Interleukin-8, *TGF-β* Transforming growth factor β, *TNF-α* tumor necrosis factor alpha

### Tension-type headache

Four cytokines were assessed in at least two studies comparing tension-type headache and healthy controls (see Table 4). There were significantly higher levels of both Transforming growth factor (TGF)-β (SMD 0.52, 95% CI 0.18–0.86, *p* = 0.003) and TNF-α (SMD 0.64, 95% CI 0.33, 0.96, *p* = 0.0001) in patients with tension-type headache. Both were only in two studies, but with similar results and low heterogeneity (I^2^ = 0%). There was no significant difference in IL-2 or IL-8 (see Table 4).

**Table 4:** Differences in circulating cytokines levels between patients with tension-type headache and healthy controls

Cytokine	N of studies	Total participants	Std. Mean Difference (95% CI) Effect Estimate	*P* Value	I^2^
IL-2	2	187	-5.48 (-14.13, 3.17)	0.21	99%
IL-8	3	242	1.08 (-1.02, 3.18)	0.31	98%
TGF-β	2	168	0.52 (0.18, 0.86)	0.003	0%
TNF-α	2	165	0.64 (0.33, 0.96)	0.0001	0%

*Abbreviations*: *CI* Confidence interval, *I*^*2*^ Measure of heterogeneity, *IL-2* Interleukin-2, *IL-8* Interleukin-8, *TGF-β* Transforming growth factor β, *TNF-α* Tumor necrosis factor alpha

### Cluster headache

Only a single cytokine (IL-1β) was compared between cluster headache and healthy controls in at least two studies, which was not significantly different (SMD 3.36, 95% CI -1.96–8.68, *p* = 0.22).

### New daily persistent headache

No studies were identified which compared cytokine levels with healthy controls or provided raw data on blood cytokine levels in NDPH.

## Discussion

We have identified higher levels of IL-6, IL-8 and TNF-α in patients with migraine compared to healthy controls, higher levels of TGF-β and TNF-α in tension-type headache compared to healthy controls, and higher levels of IL-1β during attacks of migraine compared to the interictal period. This corroborates the findings of previous narrative reviews in this area that pro-inflammatory cytokines are typically raised in migraine suggesting the presence of neuroinflammation [57–60].

A meta-analyses of cytokine levels in migraine has recently been published but included far fewer studies than the current study [61]. It found that IL-1β, IL-6, and TNF-α were all higher in patients with migraine than controls. The discrepancy between the results for IL-1β in that study and the current study is likely because it included only two studies measuring IL-1β, both of which had positive results, whereas we have included seven studies. All studies from this meta-analysis were also included in the current study.

Another systematic review has compiled the results of studies comparing cytokine levels in migraine between the ictal and interictal periods [62]. The authors did not conduct a meta-analysis, but they did observe a lack of a consistent relationship between cytokine levels during the ictal and interictal states. They did find a trend for the pro-inflammatory cytokines TNF-α and IL-6 to be higher, and the anti-inflammatory cytokine IL-10 to be decreased, in the interictal period in migraine compared to healthy controls. A study in experimentally induced migraine found that the anti-inflammatory cytokine IL-4 was downregulated during attacks, along with intercellular adhesion molecule 1, suggesting that these proteins are involved in the pathway of nitric oxide stimulated (and potentially spontaneous) migraine attacks [63, 64].

To the best of our knowledge, a systematic review or meta-analysis has not previously been used to assess cytokines in tension-type headache, cluster headache, or new daily persistent headache. Our finding of increased TNF-α in tension-type headache, similarly to migraine, may suggest either an overlap in pathophysiology between migraine and tension-type headache, or it may be a non-specific finding secondary to the chronic daily headache disorder. TGF-β, which we also found to be raised in tension-type headache is usually considered an anti-inflammatory cytokine and was the only anti-inflammatory cytokine we found to be raised in any primary headache disorder. This result should be interpreted with caution as the analysis only included two studies, however it is possible that TGF-β is elevated as a response to pain, or as a compensatory response to the elevation of one or more of the proinflammatory cytokines. Cytokines have only been compared to healthy controls in a few small studies of cluster headache. Neuroimmunological mechanisms of cluster headache have been previously proposed but require further supporting evidence [65]. Cytokines have not been compared to healthy controls in any studies of NDPH. NDPH is a primary headache disorder which often has a post-infectious onset and since its first description has been hypothesised to have an immune basis [14]. Studies are required to identify whether serum cytokine levels are altered in NDPH (especially those with a post-infectious onset) in comparison to controls and patients with chronic migraine.

Migraine is not thought to be a classical inflammatory disease, and classical clinical symptoms of inflammation or blood or cerebrospinal fluid inflammatory markers are not found. However, the trend for pro-inflammatory cytokines to be higher in patients with migraine suggests that neuroinflammation mediated by cytokines could be involved in its pathophysiology. There is human and animal evidence that the pro-inflammatory cytokines IL-1β, IL-6, and TNF-α are involved in both the initiation and persistence of pain by their direct effects on nociceptive sensory neurons, and central sensitisation [5]. The presence of neuroinflammation in migraine is supported by a neuroimaging study using PET/MRI imaging with [^11^C]PBR28 ligand (a marker of glial activation) in patients with migraine and showed increased tracer uptake in the thalamus, primary/secondary somatosensory cortices, and insular cortices compared to controls [66]. A second study using similar methodology found increased tracer uptake in the meninges and occipital parameningeal tissues in patients’ migraine with visual aura, compared to both healthy controls and those with lower back pain [67]. The authors hypothesized that meningeal inflammation may be related to cortical spreading depression which initiates migraine with aura.

A pathway linking cortical spreading depression with trigeminovascular system activation has been identified via the neuronal channel Panx1, the activation of which stimulates the production of IL-1β [58, 68]. This corresponds with our finding that IL-1β was the cytokine which was consistently raised during the ictal period of migraine. Calcitonin gene-related peptide (CGRP) is present in both peripheral trigeminal neurons and central neurons, is released upon activation of the trigeminovascular system, and CGRP blocking drugs are effective in the treatment of migraine. A COX-2 dependent pathway has been identified whereby IL-1β can induce CGRP release in trigeminal ganglia neurons, which can be blocked by indomethacin [69]. CGRP is thought to induce sterile “neurogenic inflammation” in migraine, which could further induce neuroinflammation via production of inflammatory cytokines [13, 70]. Pre-clinical studies have shown that CGRP triggers the release of cytokines from T cells [71, 72]. In patients with migraine CGRP levels have been shown to highly correlate (r = 0.94) with IL-6 levels [50].

It is important to recognise that none of the studies which have measured cytokines in primary headache disorders have been longitudinal, and they did not recruit patients prior to the onset of the headache disorder. Therefore, it is possible that the higher levels of cytokines found may be secondary to chronic pain, rather than part of the biology of the headache disorder itself. There is a large literature on the possible role of cytokines in pain disorders such as fibromyalgia [73], and psychiatric disorders, particularly depression [74], where cytokine profiles appear similar to what has been found in the headache literature, suggesting they could be a non-specific biomarkers of chronic pain or chronic stress. Alternatively, they could help explain the known association of primary headache disorders with depression and other chronic pain conditions such as fibromyalgia [75, 76].

Generally, studies of cytokines in primary headache disorder have matched patient groups by age and sex, but they have not been matched by headache frequency, severity, duration, or disability levels; or used these factors as covariates when the results are analysed. A few studies have compared cytokine levels between patients with chronic (headache on at least 15 days per month) and episodic (headache on fewer than 15 days per month) headache disorders. These have found higher IL-6 levels in chronic than episodic tension-type headache [34], higher TNF-α in chronic than episodic migraine [48, 53], and higher IL-6 and CGRP in chronic than episodic migraine [53]. These studies suggest a correlation between headache frequency and higher proinflammatory cytokine levels.

A limitation of all studies measuring peripheral cytokine levels is that the activity of cytokines is predominantly paracrine (local) rather than endocrine, therefore it is possible that measuring peripherally circulating cytokines may not be reflective of their likely site of action in headache disorders either in the brain or trigeminal afferents. For this reason, one study has measured cytokine levels in jugular venous blood during migraine attacks. This study did find similar results to those studies which have investigated peripheral cytokines—an increase in proinflammatory cytokines including IL-6 and TNF-α during the attack compared to baseline [30]. Cytokine levels have also been measured in the cerebrospinal fluid (CSF) of patients with primary headache disorders, but in too few studies to include in the current systematic review and meta-analysis. A small study of patients with NDPH and chronic migraine found that TNF-α levels in the CSF were above the normal range in the majority of patients with both NDPH and chronic migraine, but were in the normal range in most patients in the serum [77]. A CSF study in patients with migraine and tension-type headache found that IL-1ra, Monocyte Chemoattractant Protein-1 (MCP-1), and TGF-β1 were higher in the CSF of patients with both episodic tension-type headache and migraine without aura, compared to controls [78].

A limitation of all cytokine studies is that there are a multitude of factors which can affect cytokine levels, including time of day the blood is taken, site blood is taken from, speed of analysis, presence of comorbidities, medications being taken, and nutritional status of the patient. This means that cytokines are unlikely to be helpful as diagnostic biomarkers, however they may still prove helpful in determining prognosis, or influencing response to treatment. Cytokine antagonists and monoclonal antibodies against cytokines or their receptors are in clinical use for autoimmune disorders. To the best of our knowledge there are no case reports or anecdotal evidence of monoclonal antibodies targeting TNF-a, IL-6, or IL-8 improving primary headache disorders, but they have not been trialled specifically for this purpose.

## Conclusions

The proinflammatory cytokines IL-6, TNF-α, and IL-8 are higher in patients with migraine than healthy controls, and IL-1β levels are raised during migraine attacks. This suggests that they may be involved in the pathophysiology of migraine. Prospective studies are required to determine causality, and to determine whether cytokines are useful biomarkers in differentiating different subtypes of headache disorders, determining prognosis, or influencing treatment response.

## Data Availability

The extracted data used for the analyses is available upon reasonable request to the corresponding author.

## Abbreviations

CSF: Cerebrospinal fluid
ICHD-3: International Classification of Headache Disorders, 3rd Edition
IHS: International Headache Society
IFN-γ: Interferon gamma
IL: Interleukin
IQR: Interquartile range
NDPH: New daily persistent headache
SD: Standard deviation
SMD: Standardised mean difference
TGF-β: Transforming growth factor beta
TNF-α: Tumour necrosis factor alpha
